# Intraoperative discovery of bladder herniation in a known inguinoscrotal hernia during open cystolithotomy, prostatectomy and hernioraphy : a case report and review of diagnostic pitfalls

**DOI:** 10.1186/s12894-026-02269-0

**Published:** 2026-07-23

**Authors:** Hassami Sawadogo, Abdoul-Karim Paré, Abdoul-Karim Ouattara, Fatao Ouédraogo, Békaye Ouédraogo, Tiéoulé Mamadou Traoré, Clotaire Alexis Marie Kiemdiba Donega Yaméogo, Brahima Kirakoya, Adama Ouattara

**Affiliations:** 1 Department of Urology, Dedougou Regional Hospital, Dedougou, Burkina Faso; 2 Division of Urology, Souro Sanou University Teaching Hospital, Bobo-Dioulasso, Burkina Faso; 3 Division of Chirurgy, Manga Regional Hospital, Manga, Burkina Faso; 4 Division of Urology, Yalgado Ouedraogo University Teaching Hospital, Ouagadougou, Burkina Faso; 5 Division of Urology, Ouahigouya University Teaching Hospital, Ouahigouya, Burkina Faso

**Keywords:** Inguinoscrotal bladder hernia, Scrotal cystocele, Benign prostatic hyperplasia, Intraoperative discovery, Vesical calculus

## Abstract

**Background:**

Inguinoscrotal herniation of the urinary bladder (ISHB) is a rare but underdiagnosed condition, accounting for 1–4% of inguinal hernias and discovered intraoperatively in more than 80% of cases. Recognised predisposing factors include advanced age, obesity, and chronic infravesical obstruction, of which benign prostatic hyperplasia (BPH) is the most common. Failure to identify ISHB preoperatively exposes the surgeon to the risk of inadvertent vesical injury, reported in up to 12% of unprepared herniorrhaphies.

**Case presentation:**

A 61-year-old man was admitted for obstructive lower urinary tract symptoms complicated by chronic urinary retention, in association with a right inguinoscrotal swelling. Ultrasonography demonstrated a 145 g prostatic enlargement and a 43 × 27 mm vesical calculus. A combined procedure was planned: transvesical open cystolithotomy, prostatectomy, and inguinoscrotal hernia repair. During inguinal dissection, the absence of a true peritoneal sac prompted meticulous exploration, which identified the bladder as the hernial content. The bladder was safely reduced into the pelvic cavity without iatrogenic injury, and a Bassini herniorrhaphy was performed. The postoperative course was uneventful. At 10 months, the patient reported marked voiding improvement, with a maximum urinary flow rate of 18 mL/s.

**Conclusions:**

In male patients aged over 50 years presenting with an inguinal hernia in the setting of BPH, LUTS, or vesical calculus, selective preoperative imaging (CT or cystography) should be considered when clinical examination or risk factor profiling raises suspicion of bladder herniation. When the diagnosis is not established preoperatively, meticulous intraoperative dissection remains the cornerstone of safe herniorrhaphy and the principal safeguard against iatrogenic vesical injury. Simultaneous management of ISHB and coexisting urological conditions is feasible and advantageous, particularly in resource-limited settings.

## Background

Inguinoscrotal herniation of the urinary bladder (ISHB), also referred to as scrotal cystocele in the English-language literature, is a rare urological condition with an overall incidence of 1–4% among inguinal hernias in the general population. This proportion rises to approximately 10% in obese men aged 50–70 years [1, 2]. Despite its relative frequency in this high-risk subgroup, the entity remains largely underrecognised, and a preoperative diagnosis is established in fewer than 7% of cases [3, 4].

Recognised predisposing factors include male sex, advanced age, obesity, weakness of the abdominopelvic wall musculature, and conditions causing chronic infravesical obstruction, including BPH [5, 6]. In the vast majority of cases, ISHB is discovered incidentally during surgical dissection, exposing the operating surgeon to the risk of inadvertent vesical injury, a complication reported in up to 12% of herniorrhaphies performed for unrecognised bladder hernias [4].

We report a case of intraoperative discovery of ISHB in a patient undergoing combined management of voluminous BPH complicated by a vesical calculus and associated with a right inguinoscrotal hernia.

Beyond the rarity of this condition, this case highlights important diagnostic pitfalls and illustrates key intraoperative considerations that may help prevent iatrogenic bladder injury.

## Case presentation

A 61-year-old male patient was admitted for obstructive lower urinary tract symptoms of several years’ duration complicated by chronic urinary retention, in association with a right inguinoscrotal swelling. Physical examination revealed a chronic bladder globe and an uncomplicated right inguinoscrotal hernia. The diagnosis of chronic urinary retention was established on the basis of the clinically palpable bladder globe and ultrasonographic confirmation of a post-void residual volume of 340 mL.

Laboratory investigations showed a normal serum creatinine level of 102 µmol/L and a total prostate-specific antigen (PSA) of 3.5 ng/mL.

Urinary tract ultrasonography demonstrated prostatic hypertrophy estimated at 145 g and a vesical calculus measuring 43 × 27 mm (Fig. 1). The upper urinary tract appeared normal.

**Fig. 1:**
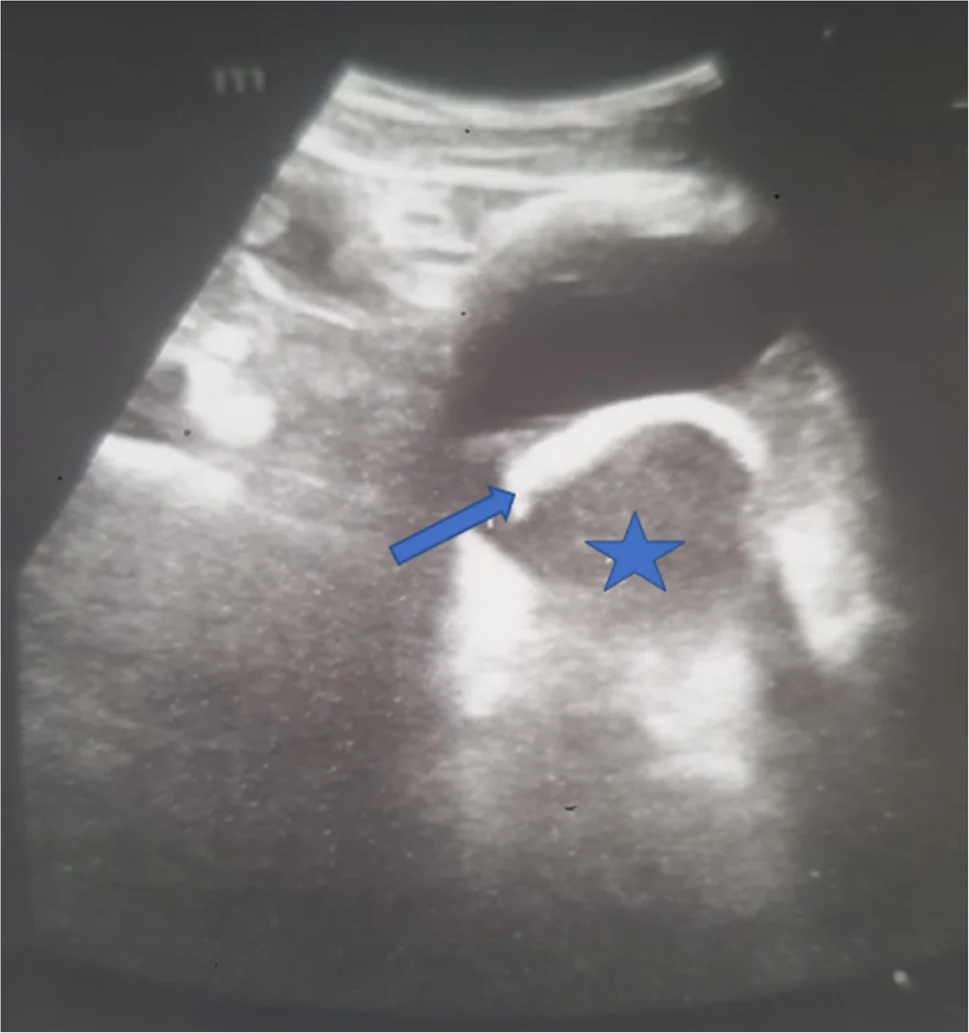
Pelvic ultrasonography demonstrating marked prostatic enlargement (asterisk) and a vesical calculus (arrow)

Semi-rigid urethrocystoscopy revealed a vesical calculus approximately 4 cm in maximum diameter (Fig. 2). The calculus was firmly adherent to the posterior bladder wall and could not be mobilized endoscopically; consequently, it persistently obscured the underlying posterior and inferolateral bladder surfaces.

**Fig. 2:**
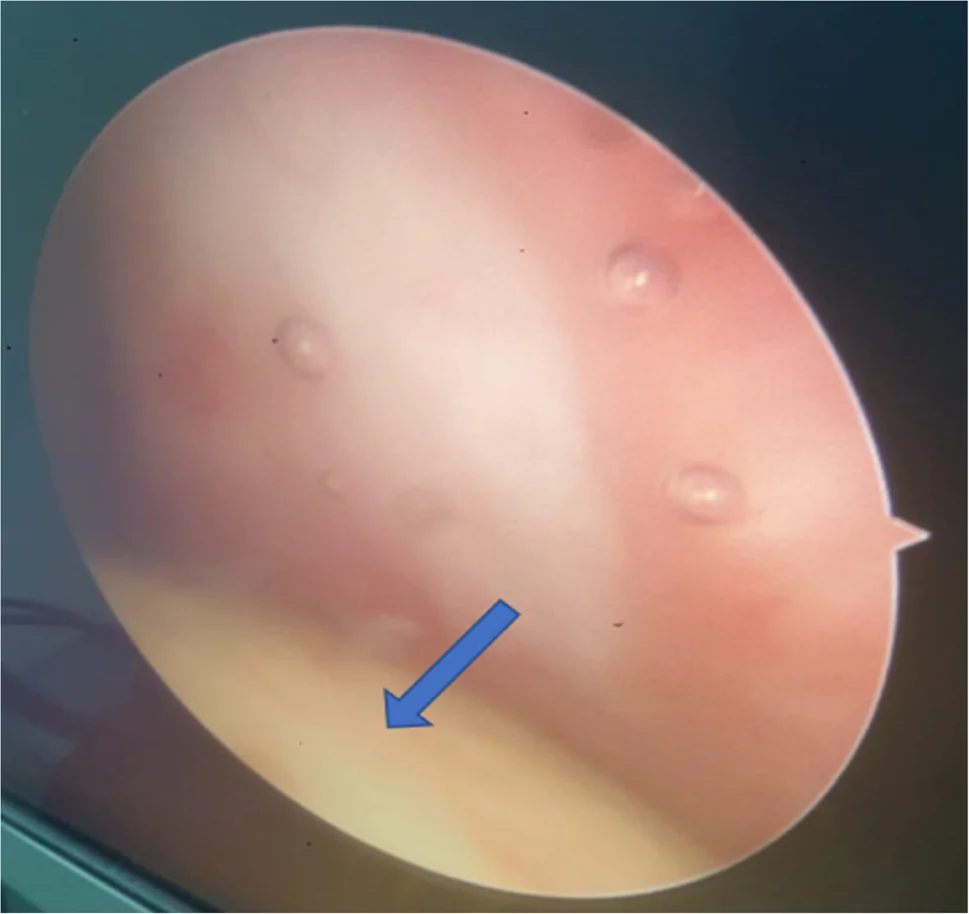
Semi-rigid urethrocystoscopy revealing a vesical calculus (arrow)

A combined procedure (cystolithotomy, transvesical open prostatectomy, and repair of the inguinoscrotal hernia) was planned due to the large stone and symptomatic voluminous BPH contributing to obstruction (beyond the stone alone).

Surgery was performed under spinal anaesthesia. Through a Pfannenstiel incision, the abdominal wall layers were opened and the bladder was approached extraperitoneally. Cystotomy was performed and a 48 mm vesical calculus adherent to the posterior bladder wall was extracted. The bladder was distended, with a thin wall and little tone upon palpation, suggestive of chronic bladder decompensation. Transvesical open prostatectomy was subsequently performed, with enucleation of a 155 g adenoma (Fig. 3).

**Fig. 3:**
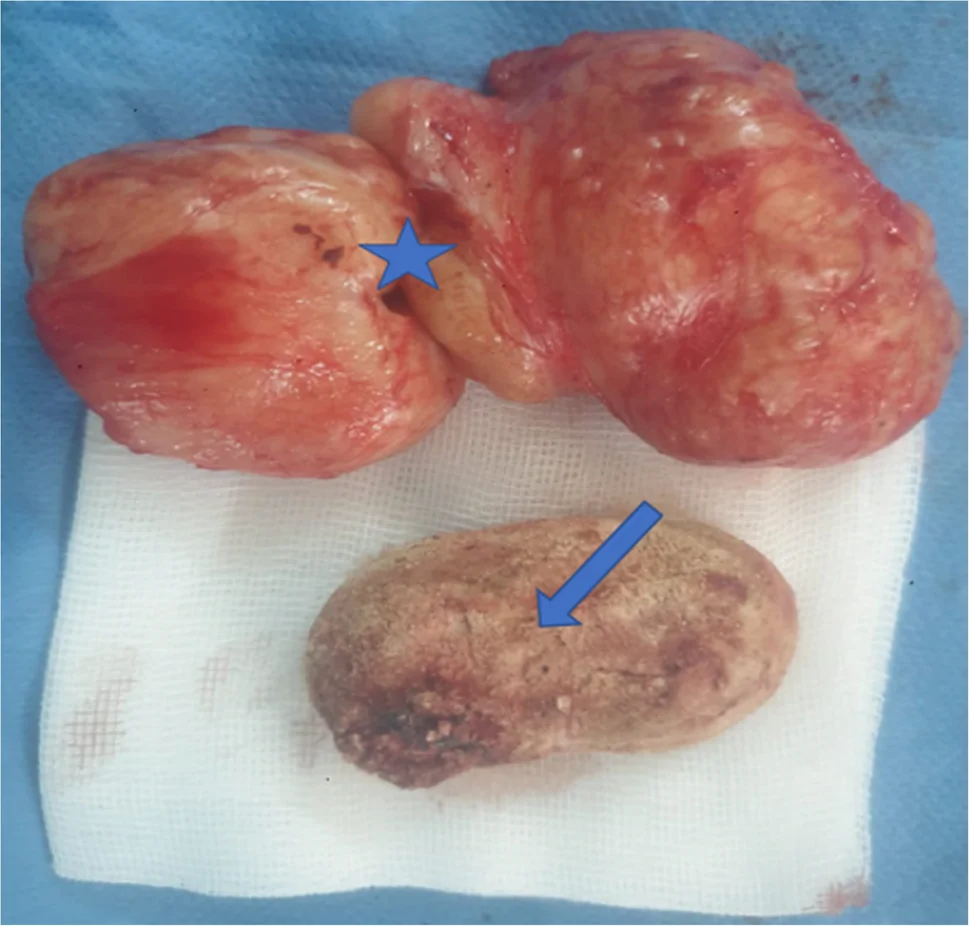
Surgical specimens including bladder stone (arrow) and prostate adenoma (asterisk)

After completion of cystolithotomy and transvesical prostatectomy through a Pfannenstiel approach, the bladder was closed. For hernia repair, a separate oblique inguinal incision was chosen rather than extending the Pfannenstiel incision laterally, to allow optimal exposure of the inguinal canal and scrotal extension while minimizing disruption to the recent bladder closure. At dissection, the absence of a true peritoneal sac prompted heightened surgical vigilance. Meticulous dissection then allowed identification of the bladder as the herniating content, engaged within the inguinal canal and extending into the scrotum (Fig. 4).

**Fig. 4:**
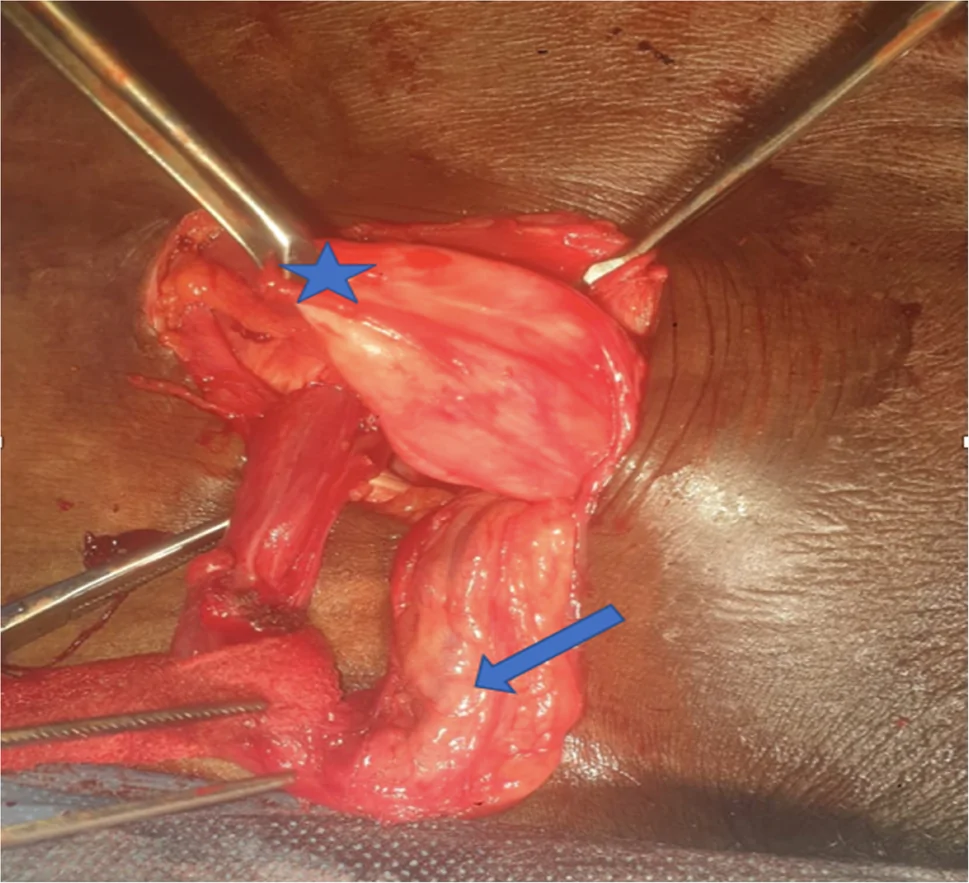
Intraoperative view showing spermatic cord (arrow) and the urinary bladder (asterisk) as the hernial content, engaged within the inguinal canal and extending into the scrotum

No iatrogenic vesical injury occurred. Following careful lysis of adhesions, the bladder was reduced into the pelvic cavity and a Bassini herniorrhaphy was performed.

The postoperative course was uneventful. The urinary catheter was removed two weeks after surgery.

Histopathological examination of the surgical specimen showed prostatic adenoleiomyoma with foci of chronic prostatitis and no evidence of malignancy.

At 10 months postoperatively, the patient reported a marked improvement in voiding. Clinical examination was unremarkable. Uroflowmetry demonstrated a maximum urinary flow rate (Qₘₐₓ) of 18 mL/s.

## Discussion

Although rare, inguinal bladder hernia remains underreported in the literature. A literature review published in 2022 identified 73 adult cases in Medline over the previous ten years, highlighting the persistent rarity and frequent underdiagnosis of this condition [7]. This case illustrates several key clinical lessons regarding ISHB and underscores the diagnostic challenge that is consistently documented across the published literature: Watson, as cited by Sarr et al., reported that 80.4% of 347 vesical hernia cases were discovered intraoperatively, and Kraft et al. confirmed that fewer than 7% were recognised before surgery [1, 6]. In our case, ISHB was identified solely by virtue of operative vigilance in response to the absence of a peritoneal sac, thereby averting a potentially serious vesical injury.

Benign prostatic hypertrophy (BPH) is a major risk factor for inguinal bladder hernia, due to the chronic increase in intravesical pressure that it causes. This association is common, as illustrated by the series by Sarr et al. who report associated BPH in 3 out of 8 cases (37.5%) [6]. Our patient cumulated several well-established risk factors: age over 50 years, voluminous BPH with chronic urinary retention, and a stasis vesical calculus. Multiple authors have emphasised the necessity of systematically considering ISHB whenever an inguinal hernia is associated with LUTS in an elderly male patient [3]. The sign of Méry (reduction of the hernial swelling following micturition, or the need to compress the scrotum to complete bladder emptying (two-stage voiding) is pathognomonic when present but remains inconstant [2, 8, 9]. In our patient, this sign was not elicited during preoperative examination, which, in combination with local resource constraints limiting routine cross-sectional imaging, contributed to the absence of a preoperative computed tomography (CT) or cystography. Had it been elicited, this sign would have prompted preoperative imaging.

Contrast-enhanced abdominopelvic CT is the reference imaging modality for confirming the preoperative diagnosis of ISHB in patients with high clinical suspicion, particularly obese men over 50 years of age presenting with LUTS [1, 3]. It precisely delineates the vesical nature of the hernial content, assesses the hernia neck, identifies associated ureteral anomalies, and guides surgical planning. Retrograde cystography demonstrates the classic ‘dog-ear’ or ‘dumbbell’ appearance of the herniated bladder [9, 10]. Ultrasonography may suggest the diagnosis by revealing a hypoechoic scrotal content that varies in volume with micturition, and represents a useful alternative in low-resource settings [10]. In our case, the absence of dedicated preoperative hernia imaging (CT or cystography) resulted in an unanticipated intraoperative finding, reinforcing the critical role of this step in operative planning. However, it should be acknowledged that routine preoperative imaging is not universally recommended for patients with inguinal hernias. Imaging strategies should be tailored to the clinical context, available resources, and local practice patterns. Consequently, cross-sectional imaging is best reserved for selected patients in whom specific clinical features raise suspicion of ISHB rather than being performed systematically.

In our case, the absence of a peritoneal sac at dissection is characteristic of an extraperitoneal vesical hernia, in which the bladder wall itself constitutes the hernia sac. This configuration carries a significant risk of iatrogenic injury during unprepared herniorrhaphy, as the bladder wall may be mistaken for the peritoneal sac [6].

When ISHB is not recognised preoperatively, systematic and methodical dissection of the hernia sac remains the most reliable safeguard against vesical injury. Several authors recommend filling the bladder via a urinary catheter as soon as the nature of the hernial content appears uncertain, in order to formally identify the vesical tissue before any division [3]. Vesical injury occurring in 12% of herniorrhaphies for undiagnosed ISHB carries serious consequences: vesicocutaneous fistula, wound infection, and operative field contamination, converting a clean procedure into a contaminated one [4, 6]. Sarr et al. reported accidental vesical injuries in their series of eight cases, necessitating cystorrhaphy and prolonged drainage [6]. In our patient, the attention paid to the absence of a true peritoneal sac enabled timely identification of the vesical hernia and safe reduction without incident.

The surgical management of ISHB is based on reduction of the herniated bladder followed by wall repair. Partial cystectomy is reserved for situations involving bladder wall necrosis, a narrow hernia neck, a herniated bladder diverticulum, or an intravesical tumour within the sac [1, 2, 10]. In all other circumstances, simple reduction combined with herniorrhaphy suffices. Wall repair may be performed without mesh, as in our case using the Bassini technique, or with prosthetic reinforcement according to the Lichtenstein technique, which is increasingly advocated for its durability and reduced recurrence rate [2, 11]. Simultaneous combination of hernia repair with additional urological procedures as in our case (cystolithotomy and open prostatectomy) is feasible and confers clear economic and anaesthetic benefits, particularly in resource-limited settings [6]. Simultaneous surgical procedures has been reported in isolated cases in resource-limited settings, but no large series exist; the present case adds to this limited experience.

When unrecognised and left untreated, ISHB may progress to severe complications. Urinary stasis within the herniated bladder segment predisposes to recurrent urinary tract infections and vesical calculus formation. In the literature, Duran et al. described in 2021 the seventh reported case of a bladder stone herniated into the scrotum [5]. Other documented complications include vesicoureteral reflux, hydronephrosis, acute renal failure due to trigonal compression, and vesical infarction secondary to strangulation at the hernia neck [1, 3, 6]. A review of 116 cases further revealed an 11% incidence of associated urogenital tumours in the setting of inguinoscrotal bladder hernias, supporting the systematic performance of preoperative cystoscopy [4]. The series by Sarr et al. highlights that in African clinical practice, ISHB is discovered intraoperatively in 75% of cases, a figure comparable to international data, yet reflecting a context in which systematic preoperative imaging has not yet become standard of care [6].

## Conclusion

Inguinoscrotal herniation of the urinary bladder is a rare but not exceptional entity, for which a preoperative diagnosis is seldom established. The present case serves as a reminder that in male patients aged over 50 years presenting with an inguinal hernia in the context of BPH, LUTS, or vesical calculus, selective preoperative imaging (CT or cystography) should be considered when clinical examination raises the suspicion of bladder herniation. Careful risk stratification should guide the decision. When the diagnosis is not made preoperatively, only careful and methodical dissection can prevent iatrogenic vesical injury and its associated complications. The simultaneous single-session management of ISHB with open prostatectomy and cystolithotomy, as demonstrated in this case, is both feasible and advantageous in resource-constrained settings.

## Data Availability

No datasets were generated or analysed during the current study.

## Abbreviations

BPH: Benign prostatic hyperplasia
CT: Computed tomography
ISHB: Inguinoscrotal herniation of the bladder
LUTS: Lower urinary tract symptoms
PSA: Prostate-specific antigen
Qmax: Maximum urinary flow rate
